# Denaturation of proteins: electrostatic effects *vs.* hydration

**DOI:** 10.1039/d2ra01167k

**Published:** 2022-03-31

**Authors:** Matthias Ballauff

**Affiliations:** Institut für Chemie und Biochemie, Freie Universität Berlin Takustraße 3 14195 Berlin Germany matthias.ballauff@fu-berlin.de

## Abstract

The unfolding transition of proteins in aqueous solution containing various salts or uncharged solutes is a classical subject of biophysics. In many cases, this transition is a well-defined two-stage equilibrium process which can be described by a free energy of transition Δ*G*_u_ and a transition temperature *T*_m_. For a long time, it has been known that solutes can change *T*_m_ profoundly. Here we present a phenomenological model that describes the change of *T*_m_ with the solute concentration *c*_s_ in terms of two effects: (i) the change of the number of correlated counterions Δ*n*_ci_ and (ii) the change of hydration expressed through the parameter Δ*w* and its dependence on temperature expressed through the parameter dΔ*c*_p_/d*c*_s_. Proteins always carry charges and Δ*n*_ci_ describes the uptake or release of counterions during the transition. Likewise, the parameter Δ*w* measures the uptake or release of water during the transition. The transition takes place in a reservoir with a given salt concentration *c*_s_ that defines also the activity of water. The parameter Δ*n*_ci_ is a measure for the gain or loss of free energy because of the release or uptake of ions and is related to purely entropic effects that scale with ln *c*_s_. Δ*w* describes the effect on Δ*G*_u_ through the loss or uptake of water molecules and contains enthalpic as well as entropic effects that scale with *c*_s_. It is related to the enthalpy of transition Δ*H*_u_ through a Maxwell relation: the dependence of Δ*H*_u_ on *c*_s_ is proportional to the dependence of Δ*w* on temperature. While ionic effects embodied in Δ*n*_ci_ are independent of the kind of salt, the hydration effects described through Δ*w* are directly related to Hofmeister effects of the various salt ions. A comparison with literature data underscores the general validity of the model.

## Abstract 2

A phenomenological approach to the unfolding transition of proteins is given. The model treats quantitatively the effect of electrostatics as well as of hydration (Hofmeister effects).

## Graphical abstract

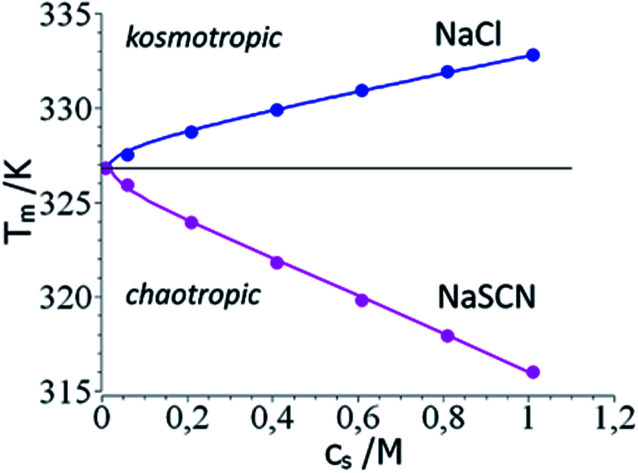

## Introduction

The denaturation of proteins by a globule to coil transition is a classical subject of biophysics.^1^ The thermal denaturation in which the protein goes from natural folded state to a random coil in aqueous solution occurs with raising temperature. Cold denaturation,^2^ which has been known for a long time, is the transition to denatured state taking place with decreasing temperature. It is well-established that for many proteins chain denaturation is a two state transition^3–6^ in which the globular and the denatured form of the protein are well-defined thermodynamic states in equilibrium with each other. Hence, an equilibrium constant *K*_u_ can be defined between the globular and denatured state which allows us to treat the denaturation as a fully thermodynamic problem relating the melting temperature *T*_m_ to the transition enthalpy Δ*H*_u_ and the transition entropy Δ*S*_u_.^7^

A fundamental problem in the field is the change *T*_m_ of a given protein with solutes in the aqueous phase. Up to now, there have been an enormous number of experimental studies that started out in the sixties of the last century.^8^ There are many investigations that study the change of *T*_m_ in the presence of various salts and non-charged solutes which can stabilize or destabilize the globular state.^4,6,7,9–20^ This effect is of obvious biological importance and can be traced back to hydration effects embodied in the Hofmeister series.^21–24^ The collapse transition of poly(*n*-isopropylacrylamide) (PNIPAM) in aqueous solution is another well-studied and fundamental problem where a coiled polymer undergoes a transition from the coiled to the globular state with raising temperature. Here too there is a large number of fundamental and detailed studies on this transition in solutions of various ions.^23,25–30^ Taken together the folding/unfolding transition of proteins and polymers in general is problem of fundamental importance.

Early studies of protein denaturation clearly revealed the central role of charge–charge interaction.^1^ The unfolding of the globular protein exposes charged groups to water and this interaction leads to an important contribution to the free energy of unfolding that scales with the logarithm of the salt concentration in solution.^1^ This term is due to the release or uptake of ions during unfolding and play an important role both for unfolding of proteins as well as for denaturing of DNA in presence of various salts (see the discussion in ref. 31). A similar process takes place when polyelectrolytes form a complex with a protein (counterion release force; see the discussions in ref. 1 and 32–34 and further citations given there). Here a wealth of experimental data demonstrates that this effect is purely entropic and therefore independent of temperature.^32,35–37^

The unfolding of proteins also exposes hydrophobic amino acids to water. As mentioned above, hydration therefore plays an important role which has been the subject of exhaustive investigations by Record and coworkers in the frame of the solute partitioning model (SPM).^22,31,38–40^ This model treats the partitioning of the solute ions or solutes between the hydrate and the bulk water. Kosmotropic ions are depleted from the hydrate water whereas chaotropic ions are enriched in this phase. Moreover, these investigations have clearly revealed that effects due to the partitioning of solutes scale linearly with salt concentration which is in full agreement with the analysis by Schellman using Kirkwood–Buff integrals.^4,5^ Thus, for many kosmotropic salts in the Hofmeister series, a linear relation between the free energy and salt concentration is found (“*m*-value”; see the discussion in ref. 29). In many cases the *m*-value is found to be independent of temperature. Based on these considerations, Chen and Schellman developed a phenomenological model that is based on a *m*-values that do not depend on temperature^6,41^ (“linear model”; *cf.* ref. 18). A fact overlooked in later expositions of this theory is the linear dependence of the specific heat Δ*c*_p_ on salt concentration. Chen and Schellman could demonstrate that this dependence is a direct consequence of the assumption of a constant *m*-value.^6^ The notion of a *m*-value independent of temperature, however, is a stringent condition that may not be fulfilled for a given system.^42^ Hence, a general model should avoid this prerequisite.

Surveying the literature on denaturation of proteins, it becomes clear that exchange of water and counterions during unfolding present two important factors that determines the stability of proteins in aqueous solution to a large extend. Both are modified by the added solute. Hence, a quantitative treatment of the effect of ions and water is a necessary prerequisite for a quantitative evaluation of data related to the unfolding of proteins in presence of various solutes. In a recent paper we have presented a unified approach for the free energy of complex formation between proteins and polyelectrolytes that comprises both effects.^34^ Temperature *T* and salt concentration *c*_s_ were identified as the decisive variables and a closed expression for the free energy Δ*G*_b_(*T*,*c*_s_) of complex formation could be derived. In this model counterion release was characterized by Δ*n*_ci_ denoting the net number of released ions during binding whereas hydration was described in terms of the parameter Δ*w* defined already in early expositions of the problem^1,43,44^ and used frequently to describe the effect of hydration on complex formation.^44–49^ Central for the development of this model is the fact that mixed derivatives of the binding enthalpy Δ*H*_b_(*T*,*c*_s_) with regard to *T* and *c*_s_ must be the same. Hence, this Maxwell-relation leads to prediction that the dependence of Δ*H*_b_(*T*,*c*_s_) on *c*_s_ gives directly the dependence of Δ*w* on temperature. The model thus derived is capable of describing the weak dependence of Δ*G*_b_(*T*,*c*_s_) on temperature which in turn leads to a strong compensation of enthalpy and entropy.^34^ Moreover, the values obtained for Δ*n*_ci_ and Δ*w* obtained by the present model for the denaturation of a given protein can directly be compared to data deriving from studies of complex formation of polyelectrolytes with proteins.^33,46,47,50,51^

Based on this model we here present a phenomenological approach to unfolding transitions of proteins that are partially charged. A closed expression for the free energy of unfolding will be presented that contains both the effect of electrostatics as well as of hydration. The consequences of the model for data evaluation will be discussed and exemplified using recent experimental data.^16,18^ The entire discussion presented here aims at a systematic analysis of experimental data obtained on polymeric unfolding transitions of various systems in aqueous phase.

## Theory

We consider the transition of a single chain of a polypeptide from a folded to an unfolded state in sufficiently dilute solution. In each stage of this transition the unfolded state is in equilibrium with the still folded part of the chain. This two-state mechanism is well-established for a great number of systems (see the discussion of this point in ref. 1, 16 and 18). Experimentally, the unfolding transition can be monitored *e.g.* by measurements of the circular dichroism leading to a fraction *α* of unfolded protein. The equilibrium constant *K*_u_ for the process of unfolding is related to *α* by1
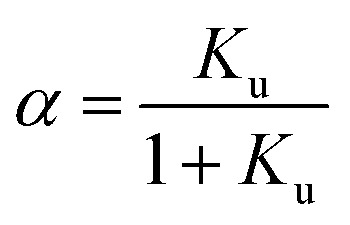
whereas the free energy of unfolding Δ*G*_u_ is related to *K*_u_ through

2 Δ*G*_u_ = −*RT* ln *K*_u_

The basic thermodynamic analysis Δ*G*_u_ was already discussed a long time ago by Record, Anderson, and Lohman.^1^ In general, the change of the equilibrium constant *K*_u_ with the activity *a*_±_ of an added salt is given by3
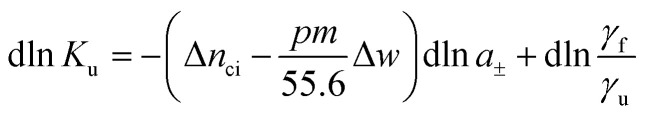
where Δ*n*_ci_ denotes the total number of released or taken-up ions during the process of unfolding. The parameter Δ*w* treats the release or uptake of water in the course of the unfolding transition while *p* = 2 for monovalent salt with molality *m*. By definition, Δ*w* is independent of salt concentration. The factor 55.6 is the molality of water and the parameters *γ*_f_ and *γ*_u_ are the activity coefficients of the chain in the folded and unfolded state, respectively. Note that this equation with necessary adaptions has been the basis of our recent discussion of complex formation of polyelectrolytes with proteins.^34^ In the following, the same approximations will be made: (i) the change of the activity coefficients *γ*_f_ and *γ*_u_ with the activity *a*_±_ of the added salt give a small but non-negligible contribution of the term Δ*n*_ci_ (see the discussion in ref. 1), (ii) the mean activity coefficient of the salt ions will be set to unity, and (iii) the molality *m* of the salt will be equated to its concentration *c*_s_. With these approximations the justification of which will be discussed below eqn (3) becomes4
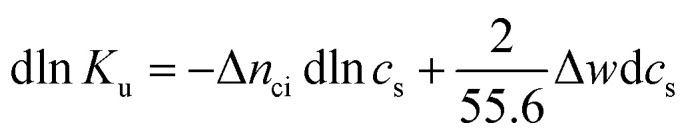


Hence, the salt concentration *c*_s_ is the variable on which the subsequent thermodynamic analysis is based. With the standard thermodynamic relation (∂ln *K*_b_/∂*T*)_*c*_s__ = Δ*H*_b_/*RT*^2^ we obtain the differential of ln *K*_u_ for monovalent ions5
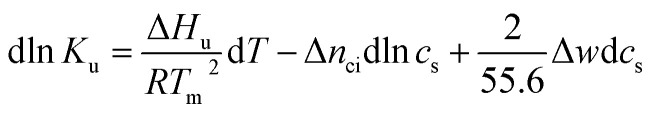
where Δ*H*_u_ denotes the enthalpy change at the unfolding transition and *T*_m_ the respective temperature of unfolding. Thus, in the following the unfolding transition will be treated as the function of the two decisive variables, namely temperature *T*_m_ and salt concentration *c*_s_.

There is abundant experimental evidence that the parameter Δ*n*_ci_ is independent of temperature.^32,34,35,37,52–54^ It is therefore safe to disregard the dependence of this parameter on *T*_m_. With this assumption and6
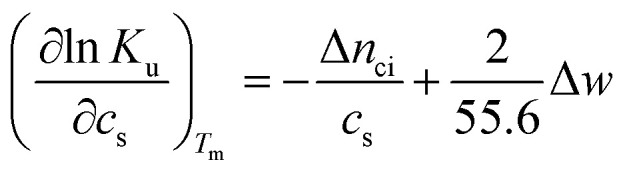
we obtain the Maxwell-relation^34^7
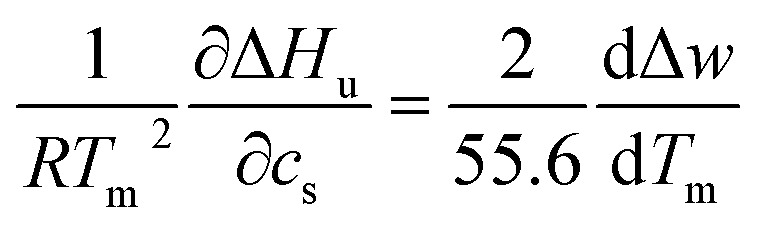


This relation demonstrates that the salt dependence of transition enthalpy is directly related to the dependence of the parameter Δ*w* on temperature. As already lined out previously,^34^ this relation can now be used to calculate Δ*w* as the function of temperature. In general, the transition enthalpy Δ*H*_u_ as the function of the melting temperature *T*_m_ and *c*_s_ can be rendered as^34^8
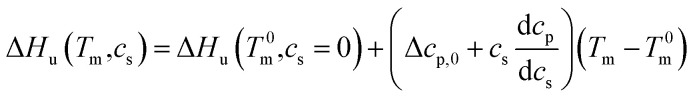
Here, the quantity Δ*c*_p,0_ denotes the change of the specific heat in absence of added salt whereas the coefficient d*c*_p_/d*c*_s_ describes the change of the specific heat with salt or solute concentration.^34^*T*^0^_m_ denotes the melting temperature for salt-free solutions. Together with eqn (7), this relations leads to9
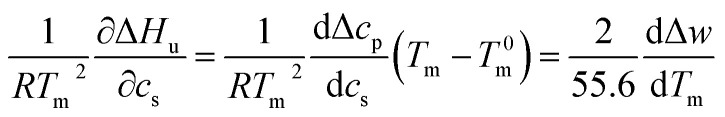


Integration leads to^34^10
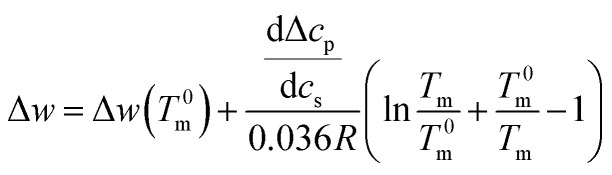
where the quantity Δ*w*(*T*^0^_m_) denotes the magnitude of Δ*w* at *T*^0^_m_ in salt-free solution.

As already discussed previously,^34^ Δ*w* can be interpreted in terms of the solute partitioning model as follows. Both the polyelectrolyte as well as the protein are hydrated in aqueous solution. During the unfolding a certain number Δ*n*_w_ of water molecules of both reactants is taken up or released. Furthermore, it is assumed that there is a partitioning of the ions between the bulk solution and the hydration water on the surface of the protein described by the partition coefficient *K*_p,+_ = (*m*^loc^_+_/*m*^bulk^_+_) for the cations where *m*^loc^_+_ denotes the molality of the cations in the hydrated shell whereas *m*^bulk^_+_ is the respective quantity in bulk. The partition coefficient *K*_p,−_ of the anions is defined in the same way. With these definitions, Δ*w* can be rendered by^34^11
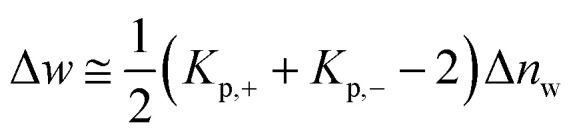


Evidently, the quantity Δ*w* measures the effect of water release on the free energy of unfolding and should not be confused with the total number Δ*n*_w_ taken up or released during unfolding. For an equal distribution of the ions between the hydrate and the bulk phase, this contribution will vanish.

In the following, we first consider uncharged systems, that is, Δ*n*_ci_ = 0. Integration of eqn (6) at constant temperature then leads to

12 ln *K*_u_ = ln *K*^0^_u_ + 0.036Δ*wc*_s_

where *K*^0^_u_ is the equilibrium constant in salt-free solution. Therefore

13 Δ*G*_u_ = Δ*G*^0^_u_ − 0.036*RT*_m_Δ*wc*_s_

Here, Δ*G*^0^_u_ denotes the free energy of unfolding at *c*_s_ = 0. Hence, the dependence of Δ*G*_u_ on *c*_s_ can be written down as14
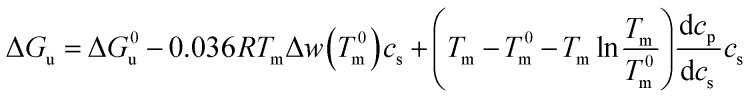


In many cases the difference *T*_m_ − *T*^0^_m_ does not exceed 10 degrees so that the last term in eqn (14) can be expanded to yield (see the derivation of eqn (11) of ref. 55)15
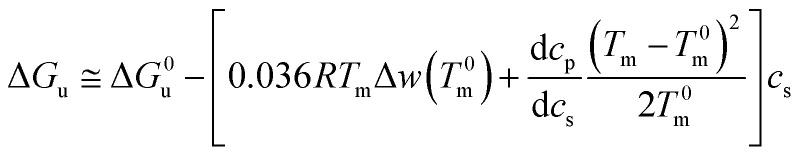


Eqn (14) may be used to calculate the *m*-value defined as the derivative of the free energy with regard to solute concentration at constant temperature16
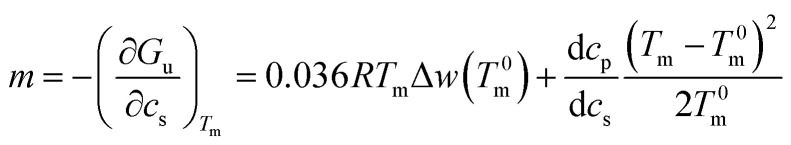


This expression shows that *m* is given by a constant plus a term that depends quadratically on *T*_m_ − *T*^0^_m_. For small temperature differences the second term will be small and the *m*-value is a constant in good approximation. However, it should be noted that *m* is in general a quantity that depends explicitly on temperature.

Eqn (14) and (15) contain only the dependence of the free energy on *c*_s_. The quantity Δ*G*^0^_u_ for salt- or solute-free solutions can be derived following the prescription of Chen and Schellman:^6^ the specific heat Δ*c*_p,0_ measured in solute-free systems can be regarded as a constant throughout the rather small temperature range under consideration here. Thus, for solute-free systems we obtain17
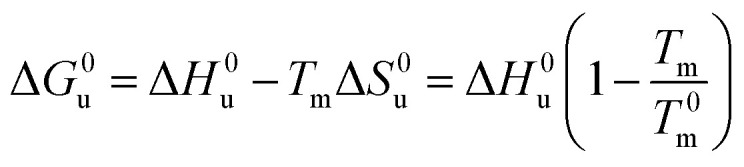
and

18 Δ*H*^0^_u_(*T*_m_) + Δ*c*_p,0_(*T*_m_ − *T*^0^_m_)

19
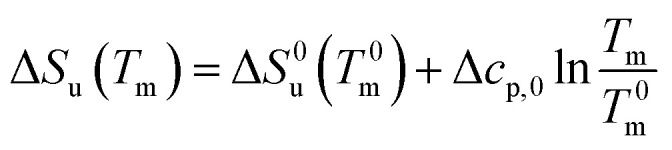
which gives20
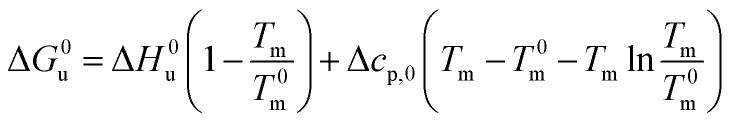


Combination with eqn (14) then leads to21
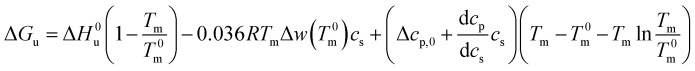


For *T*_m_ − *T*^0^_m_ ≤ 10 K this expression can be approximated by22
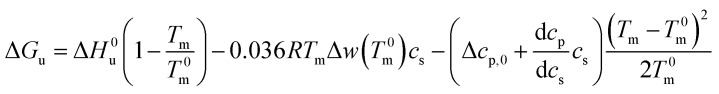


Eqn (21) and (22) are the final result for the free energy of unfolding for uncharged systems.

For partially charged proteins eqn (5) shows that a term scaling with ln *c*_s_ must be added to eqn (21).^1^ Here it must be kept in mind that there is always a small but finite salt concentration *c*_s,0_ so that the integration of eqn (5) must start at this concentration. Keeping in mind that Δ*n*_ci_ does not depend on temperature we immediately obtain from eqn (22)23
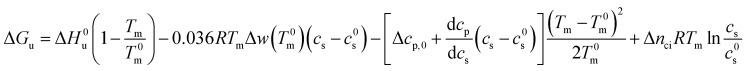


In many cases the concentration *c*^0^_s_ is small and can be disregarded in eqn (23) except for the last term, of course. Eqn (23) also shows that for small concentrations *c*^0^_s_ the free energy of unfolding may contain an appreciable contribution originating from the release or uptake of ions during denaturation. Hence, Δ*G*_u_ will be dominated by the last term for small *c*_s_. The respective transition enthalpy is given by eqn (8) where *c*_s_ is replaced by *c*_s_ − *c*_s,0_. The transition entropy follows as24
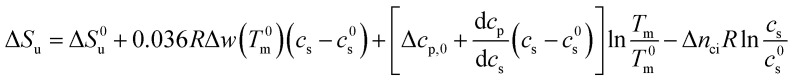


In many cases it is only possible to deduct the change of the free energy of unfolding with increasing solute concentration. Thus, we require the quantity ΔΔ*G*_u_ which gives the change of Δ*G*_u_ with *c*_s_ calculated for the transition temperature *T*^0^_m_ in solute-free solution:25
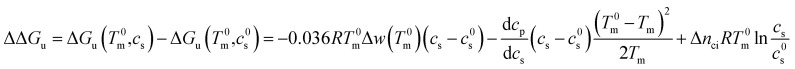


It is interesting to compare eqn (21) and (23) to phenomenological approach of Chen and Schellman^6^ (*cf.* also ref. 41). The generalized van't Hoff equation used by these authors is based on eqn (17)–(19). Moreover, the dependence of the free energy of unfolding is assumed to be linear in *c*_s_ as derived above in eqn (13):

26 Δ*G*_u_(*c*_s_) = Δ*G*^0^_u_ − *RT*_m_Δ*β*_23_*c*_s_

Thus, the coefficient Δ*β*_23_ is identical to 0.036Δ*w* in eqn (13). In the linear model of Chen and Schellman,^6^ this linear dependence has be deduced from experiments whereas the above considerations leading to eqn (13) demonstrate that this relation is a direct consequence of eqn (1). Based on these premises Chen and Schellman formulate ln *K*_u_ as follows in the present notation as:^6^27
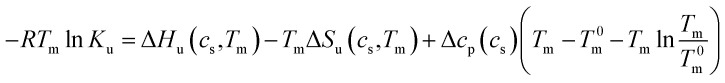
where all thermodynamic quantities Δ*H*_u_, Δ*S*_u_, are explicit function of solute concentration and temperature whereas Δ*c*_p_ is only a function of *c*_s_. All parameters will be treated as adjustable parameters for each *c*_s_ in a comparison with experimental data. The present approach, on the other hand, reveals the interrelation between the various quantities and the concentration of solute which is based on the Maxwell-relation eqn (7).

The experimental data are described in terms of 3 adjustable parameters: (i) Δ*w*(*T*^0^_m_) which is closely related to the classical *m*-value through eqn (15); (ii) the specific heat Δ*c*_p,0_ in absence of solutes; and (iii) the parameter d*c*_p_/d*c*_s_ describing the dependence of Δ*c*_p_ on *c*_s_. This parameter has been introduced by Chen and Schellman as well (the parameter 
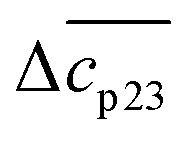
 in eqn (8) and (9) of ref. 6) but not used further. Its application to complex formation of polyelectrolytes with proteins has been discussed recently.^34^ The first two parameters are directly measurable and have an obvious physical meaning. The newly introduced parameter d*c*_p_/d*c*_s_ describes the dependence of hydration effects on temperature.

A comprehensive phenomenological analysis of the denaturation temperature for uncharged polymers was presented some time ago by Heyda and Dzubiella.^29^ Here, the hydration effects are described in terms of the preferential interaction parameter Δ*Γ*_23_. If this parameter does not depend on *c*_s_, it follows directly that
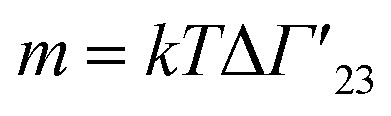
where 
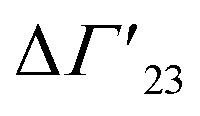
 is defined as the preferential interaction parameter independent of *c*_s_. The analysis of the changes effected by kosmotropic salts showed indeed that this equation provides a very good approximation of the experimental data obtained for the collapse transition of PNIPAM-chains in aqueous solution.^26,29^ Thus, these data could be compared directly to the prediction of the SPM with moderate success (*cf.* Table 3 of ref. 29). Moreover, Heyda and Dzubiella could estimate the entropic limit of the preferential interaction parameter 
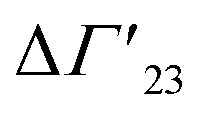
 resulting for a total exclusion of the kosmotropic ions from the surface of the unfolded protein. In this case 
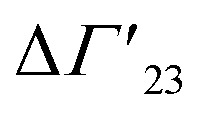
 ≅ −Δ*V* with Δ*V* being the change of the volume inaccessible for kosmotropic ions upon unfolding the protein. This parameter can be estimated from the change of the solvent accessible surface area (SASA) effected by unfolding and a length parameter *l* (∼ 0.1 nm) describing the thickness of the layer inaccessible for the ions. The estimate of the *m*-value derived from this calculation compares favorably with the measured values (*cf.* Table 3 of ref. 29). In this limit, the *m*-value (see eqn (16)) becomes independent of temperature and the *K*_p,±_ as defined through eqn (11) are much smaller than unity. If, on the other hand, the *K*_p,±_ are approximately unity, the *m*-value will be small but exhibit a considerable dependence on temperature (*cf.*eqn (11)). In this situation the dependence of the free energy of unfolding should depend quadratically on Δ*T*_m_ which has been found previously for the complex formation of polyelectrolytes with proteins.^34^ It should be kept in mind, that these considerations disregard the counterion release term in eqn (23). The *m*-value observed for charged systems where Δ*n*_ci_ ≠ 0, will differ considerably and the predictions of the SPM are related only to the parameter Δ*w* as defined through eqn (10).

In principle, eqn (23) and eqn (26) define stability curves as defined by Becktel and Schellman^3^ inasmuch as they describe the free energy Δ*G*_u_ as the function of temperature and salt concentration. If Δ*c*_p,0_ may be regarded as constant throughout a temperature range of sufficient width, the present approach could be used to construct Δ*G*_u_(*T*,*c*_s_) for all pertinent temperatures ranging from cold to thermal denaturation. Given the fact, however, that Δ*c*_p,0_ depends on temperature,^7^ such stability curves should be regarded with caution.

## Results and discussion

### Basic predictions of the model

The basis of the present model is eqn (5) which is general except for the neglect of the activity coefficients of the solute. Previous discussions, however, have shown that this approximation is inconsequential and will only change slightly the resulting parameters.^4,33,34^Eqn (5) or its integrated from has been used very often to analyze the release of water upon complex formation of highly charged macromolecules as *e.g.* DNA with various proteins.^33,46,47,50,51^ It is thus interesting to compare its magnitude for complex formation with values deriving from protein unfolding. Evidently, the parameter Δ*w* introduced by this equation does not give the number of released water molecules defined as Δ*n*_w_ but measures the thermodynamic effect of this release (see the discussion of eqn (11) above).^34^

A next prerequisite is the independence of Δ*n*_ci_ on temperature. As already mentioned above, this fact is well-borne out of a large bulk of experimental data and can safely be assumed here as well (see *e.g.* the discussion by Privalov *et al.*^32^ and in ref. 34, 37 and 52–54). This fact allows us to use the Maxwell-relation eqn (7) for the next step in which the salt dependence of the unfolding enthalpy Δ*H*_u_ is related to the dependence of the parameterΔ*w* on temperature given through eqn (9). Hence, if Δ*H*_u_ turns out to depend on the concentration *c*_s_ of the solute, it necessarily follows that Δ*w* is not a constant but depends on temperature. This fact is one of the central points inasmuch it shows that in this case the *m*-value given here by eqn (16) contains a term depending quadratically on the difference *T*_m_ − *T*^0^_m_.

The above model hence makes the following predictions that can compared directly to experiments:

(1) In a first step of the analysis of experimental data, dependence of Δ*H*_u_ on salt concentration *c*_s_ can be checked. Eqn (8) demonstrates that this quantity is a function of temperature and salt concentration *c*_s_. Moreover, the dependence of Δ*H*_u_ on salt concentration *c*_s_ gives the dependence of the quantity Δ*w* on temperature as shown by the Maxwell-relation in eqn (7) which in turn leads to the dependence of the *m*-value on temperature eqn (16). Evidently, if Δ*H*_u_ is found to depend on salt concentration, there must be a finite dependence of *m* on temperature as well (eqn (16)). If, on the other hand, the dependence of Δ*H*_u_ on salt concentration *c*_s_ is small, the parameter d*c*_p_/d*c*_s_ ≅ 0 and the terms in eqn (23) and (25) depend only on *T*_m_, that is, the quadratic term can be dismissed. Hence, the evaluation of experimental data can begin by a critical check of Δ*H*_u_(*T*,*c*_s_).

(2) The term scaling with ln *c*_s_ will profoundly change the dependence of the free energy on salt concentration and this dependence will be most marked for small *c*_s_ (*cf.*eqn (6)). The dependence of *T*_m_ on *c*_s_ will therefore be non-linear at small *c*_s_ if Δ*n*_ci_ assumes a finite value. Since the effect embodied in this parameter is of entirely entropic origin, the non-linear dependence on *c*_s_ thus effected should be independent of the nature of the added salt of same valency, that is, *T*_m_ should be a universal function of *c*_s_ for small *c*_s_. Hofmeister effects are expected to come into play only for higher salt concentrations where ΔΔ*G*_u_ scales linearly with *c*_s_. Hence, *T*_m_ is expected to be independent on the nature of the salt ions if the salt concentration is small. The observation of this effect, however, requires a small *c*_s,0_ and precise measurements at concentrations only slightly larger than *c*_s,0_. Evidently, the ionic effect embodied in Δ*n*_ci_ and the change of *T*_m_ by hydration may cancel each other. Thus, if Δ*n*_ci_ < 0 as well as Δ*w* < 0, eqn (23) demonstrates that can lead to Δ*T*_m_ = 0 for a finite salt concentration. This problem has already been discussed by Chudoba *et al.*^30^ and is seen directly in the study of the unfolding of RNase A.^16^ Similar observations have also been made for thermophilic proteins.^56,57^ The present theory allows us to model this effect in terms of the parameters Δ*n*_ci_ and Δ*w*.

(3) If the term quadratic in eqn (23) and (25) can be disregarded, that is, for small Δ*T*, the combination of both expressions shows that in this case28
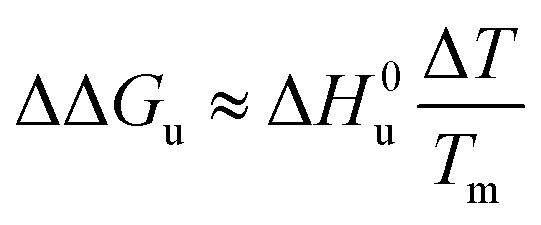
which predicts that ΔΔ*G*_u_/Δ*H*^0^_u_ ≈ Δ*T*/*T*_m_ should be an universal function. Hence, for small Δ*T*, this equation may be used to check the internal consistency of data (*cf.* the discussion of this point by Senske *et al.*^16^).

### Evaluation of data

Thus, the evaluation of the experimental data should proceed in the following steps: The unfolding is usually determined by microcalorimetric studies in which the heat change during this process is measured precisely. These measurements yield the heat of transition Δ*H*_u_(*c*_s_) at different concentrations of the solute *c*_s_ and the melting temperature *T*_m_ at the respective salt concentration *c*_s_ (see *e.g.* the discussion in ref. 15 and 18). In the following, the comprehensive set of data of Francisco *et al.*^18^ on the unfolding of ribonuclease A in presence of sodium salts will be used to exemplify the steps of evaluation. Here, the unfolding of RNase A has been observed at a pH of 4 in 10 mM acetate buffer. Therefore, the concentration *c*_s,0_ = 0.01 M in the subsequent analysis.

**Fig. 1:**
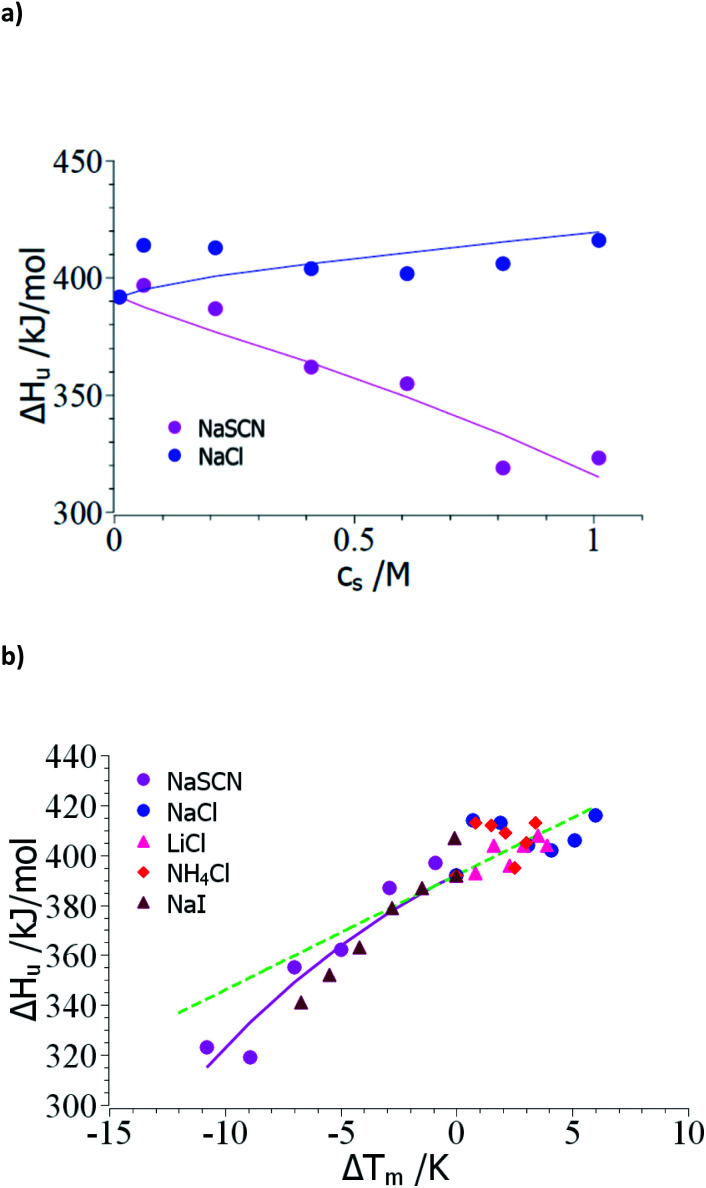
Evaluation of the measured transition enthalpy Δ*H*_u_ by eqn (8). (a) Δ*H*_u_ as the function of salt concentration *c*_s_. The marks show the experimental data for the unfolding of ribonuclease in presence of the salts indicated in the graph. These data have been taken from Table 1 of Francisco *et al.*^18^ (b) Δ*H*_u_ as the function of Δ*T*_m_ = *T*_m_ − *T*^0^_m_. The solid lines indicate the fit of eqn (8) whereas the green dashed line indicates the transition enthalpy calculated by eqn (8) with the average value Δ*c*_p,0_ ≅ 4.6 kJ (K^−1^ mol^−1^) and dΔ*c*_p_/d*c*_s_ = 0. See text for further explanation.

As outlined above, the analysis may start by the check of the dependence of Δ*H*_u_ on *c*_s_ (see Table 1 of ref. 18). Fig. 1a displays Δ*H*_u_(*c*_s_) for a typical kosmotropic salt as NaCl as well as for NaSCN which provides a good example for a chaotropic system. The enthalpy of denaturation in presence of NaCl hardly depends on salt concentration whereas a marked dependence is found for NaSCN. This test splits up the experimental data sets into two classes:

(1) Small Δ*T*_m_; kosmotropic ions: the small dependence of Δ*H*_u_ on *c*_s_ suggests that the coefficient dΔ*c*_p_/d*c*_s_ in eqn (15), (23) and (25) can be safely neglected and the only relevant parameters are Δ*n*_ci_ and Δ*w*(*T*^0^_m_). Moreover, the changes Δ*T* = *T*_m_ − *T*^0^_m_ are rather small so the term quadratic in Δ*T* in eqn (23) can hardly be determined. However, this does not imply that this term is zero for kosmotropic salts in general.

(2) Large Δ*T*; chaotropic ions: for NaSCN there is a marked dependence of Δ*H*_u_(*c*_s_) on salt concentration which immediately demonstrates that dΔ*c*_p_/d*c*_s_ assumes a finite value and the *m*-value (eqn (16)) in turn depends on temperature. Moreover, the observed Δ*T* is much larger than in case of the kosmotropic ions. Hence, fits must take into account all terms in eqn (25).

Case (1): small Δ*T*_m_; kosmotropic ions: Fig. 1b gathers all data of the enthalpy Δ*H*_u_ as the function of the difference *T*_m_ − *T*^0^_m_. The error of these numbers is of appreciable magnitude and only allows us to obtain an estimate for Δ*c*_p,0_ for which an evaluation for the data of all kosmotropic ions (NaCl, NH_4_Cl, LiCl) gives an estimate Δ*c*_p,0_ ≅ 4.6 kJ (K^−1^ mol^−1^) which compares well literature (see ref. 7 and 58). Hence, the subsequent evaluation is based on dΔ*c*_p_/d*c*_s_ = 0.

**Fig. 2:**
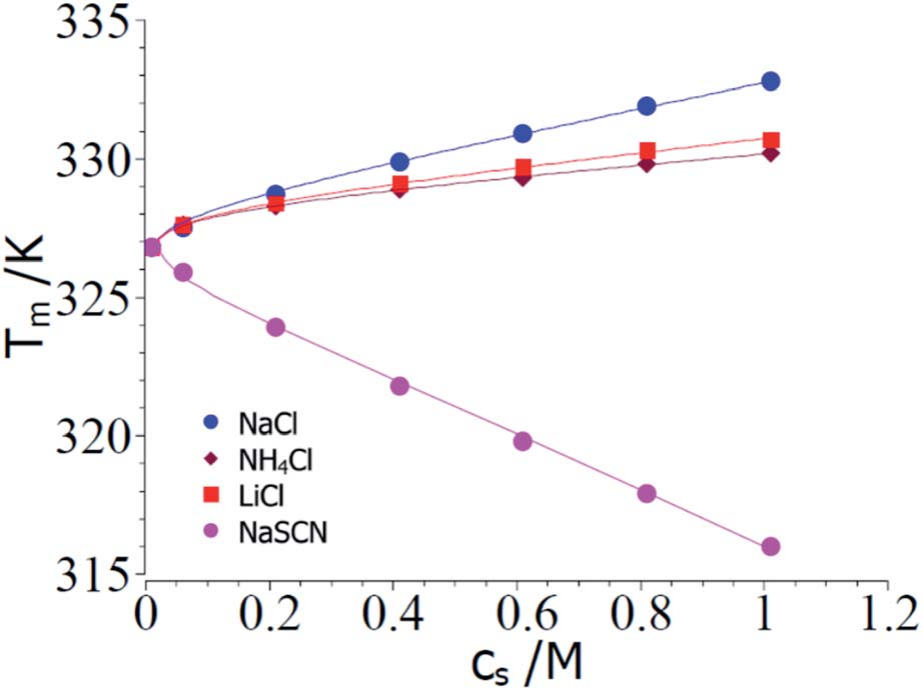
Comparison of theory and experimental data taken from the denaturation of RNase A for the 3 kosmotropic salt NaCl, NH_4_Cl, LiCl and for the chaotropic salt NaSCN.^18^ The points show the transition temperatures taken in presence of different salts as indicated in the graph (see Table 1 of ref. 18). The solid lines mark the calculated transition temperatures *T*_m_ calculated from the fit parameters Δ*n*_ci_ and Δ*w*(*T*^0^_m_) (*cf.*Table 1). See text for further explanation.

Fig. 2 displays a comparison of the experimental transition temperatures *T*_m_ as the function of salt concentration obtained by numerical solution of eqn (23) for Δ*G*_u_ = 0. Here the data *T*_m_(*c*_s_) obtained for a given salt are fitted to eqn (23) with neglect of the term quadratic in Δ*T* using the MathLab routine cftool (MATLAB (2021b). Natick, Massachusetts: The MathWorks Inc.). All calculations have been done using the value of the transition enthalpy in salt-free systems Δ*H*^0^_u_ = 392 kJ mol^−1^ and the transition temperature *T*^0^_m_ = 326.8 K given by Francisco *et al.*^18^ As mentioned above, the buffer added to all solutions leads to a *c*_s,0_ = 0.01 M.^18^ The solid lines in Fig. 2 display the respective fits whereas Table 1 gathers the respective fit parameters. A single value of parameter Δ*n*_ci_ turned out to describe Δ*G*_u_ for all systems under consideration here in agreement with the above general considerations. This fact has already been observed by Francisco *et al.*^18^ and the presence analysis compares well with eqn (21) of ref. 18 inasmuch *T*_m_ can be described by the combination of a linear and a logarithmic term (see eqn (23)). Pegram *et al.* also found that a single parameter was sufficient to describe the dependence of the unfolding of DNA as well as for the DNA-binding domain of the lac repressor at small salt concentrations.^31^ Hence, an important prediction of the present model is fully corroborated by the experimental data and the parameter Δ*w*(*T*^0^_m_) can be compared to data obtained for complex formation of polyelectrolytes with proteins.

**Table 1:** Summary of the parameters deriving from the fits of ΔΔ*G*_u_^a^

System	Δ*n*_ci_	Δ*w*(*T*^0^_m_)	dΔ*c*_p_/d*c*_s_
NaCl	0.17	−50.4	0
LiCl	0.17	−26.2	0
NH_4_Cl	0.17	−19.4	0
NaSCN	−0.17	103	2.5

a Δ*n*_ci_: number of ions released or taken up during unfolding (eqn (3) and (4)); Δ*w*: effect of water release or uptake (eqn (3) and (4)); dΔ*c*_p_/d*c*_s_: parameter describing the dependence of Δ*w* on temperature (eqn (8) and (10)).

The parameter Δ*n*_ci_ is positive for all kosmotropic salt analyzed herein. This finding points to the fact that a small but finite number of ions attached closely to the surface of the protein is released during the unfolding transition. With increasing *c*_s_ these ions are released into a reservoir with increasing activity which requires additional free energy during the unfolding transition. Hence, this effect stabilizes the folded state and leads to a higher transition temperature.

The parameter Δ*w*(*T*^0^_m_) is negative which means that the water molecules needed for the hydration of the unfolded protein must have a higher activity as the bulk water since addition of these salts increases the magnitude of Δ*G*_u_. Hence, free energy is needed to transport water from a state of lower activity in bulk to a state of higher activity in the hydrate shell upon unfolding of the protein. This effect is due to a partial depletion of these kosmotropic ions from the hydrate shell of the protein and leads to a stabilization of the folded state. The magnitude of Δ*w*(*T*^0^_m_) found here is in the same range as found previously for complex formation of proteins with DNA.^47^

It should be noted that the present analysis not only treats Δ*G*_u_ but also Δ*H*_u_ at the same time. Thus, the independence of the *m*-value of temperature follows here from an analysis of the latter quantity. Only this analysis allows us to disregard the term in eqn (25) that depends quadratically on Δ*T*^2^.

Case (2): large Δ*T*; chaotropic ions: in the following, the evaluation of the respective parameters will be shown using the data for NaSCN (Table 1 of ref. 18). Fig. 1b shows experimental Δ*H*_u_(*c*_s_) as the function of Δ*T*_m_ whereas the solid lines displays the fit of these data according to eqn (8). This fit can be stabilized by using the experimental value Δ*H*_u_(*c*_s_ = 0) = 392 kJ mol^−1^ and the specific heat Δ*c*_p,0_ = 4.6 kJ (K^−1^ mol^−1^) estimated from the analysis of the kosmotropic systems shown in Fig. 1a. For NaSCN we obtain for the parameter dΔ*c*_p_/d*c*_s_ a value of *ca.* 2.5 kJ (K^−1^ mol^−1^ M^−1^). Evidently, the small range of data and the finite accuracy of the data allows for an estimate of these quantities only. However, since these parameters present only corrections in eqn (25) and (23) and not leading terms, this error is inconsequential for the purpose at hand.

In the next step, the parameters Δ*c*_p,0_ = 4.6 kJ (K^−1^ mol^−1^) and dΔ*c*_p_/d*c*_s_ = 2.5 kJ (K^−1^ mol^−1^ M^−1^) are introduced into eqn (23) and the values of Δ*n*_ci_ and Δ*w*(*T*^0^_m_) are derived from a numerical solution of this equation for Δ*G*_u_ = 0. Input parameters are the measured *T*_m_ measured for different NaSCN-concentrations marked by points in Fig. 2. Table 1 again gathers the data obtained from this fit whereas the solid lines in Fig. 2 displays *T*_m_ calculated with the parameters Δ*c*_p,0_ = 4.6 kJ (K^−1^ mol^−1^), dΔ*c*_p_/d*c*_s_ = 2.5 kJ (K^−1^ mol^−1^ M^−1^) and the values of Δ*n*_ci_ and Δ*w*(*T*^0^_m_). Again, a full description of the experimental transition temperatures is achieved. For the chaotropic salt NaSCN the parameter Δ*w*(*T*^0^_m_) assumes a positive value which is directly related to the fact that SCN^−^-ions are adsorbed on the unfolded protein chain thus lowering the activity of the hydrate water molecules. Hence, free energy is gained when hydrating the unfolded chain by bulk water having a higher activity. The parameter Δ*n*_ci_ now has assumed a negative value. This finding points to a much stronger interaction of such chaotropic ions with the unfolded protein chains. Thus, Fang and Furo could show that chaotropic ions can associate to PNIPAM chains with a Langmuir-type association behavior while NaCl is only weakly adsorbed.^59^ This effect measured through careful measurements of the electrophoretic mobility was found strongest for SCN^−^-ions. Hence, adsorption of chaotropic ions can diminish or even reverse the effective charge of unfolded proteins. However, further investigations of *T*_m_ at very low ion concentrations are needed to clarify this problem.

**Fig. 3:**
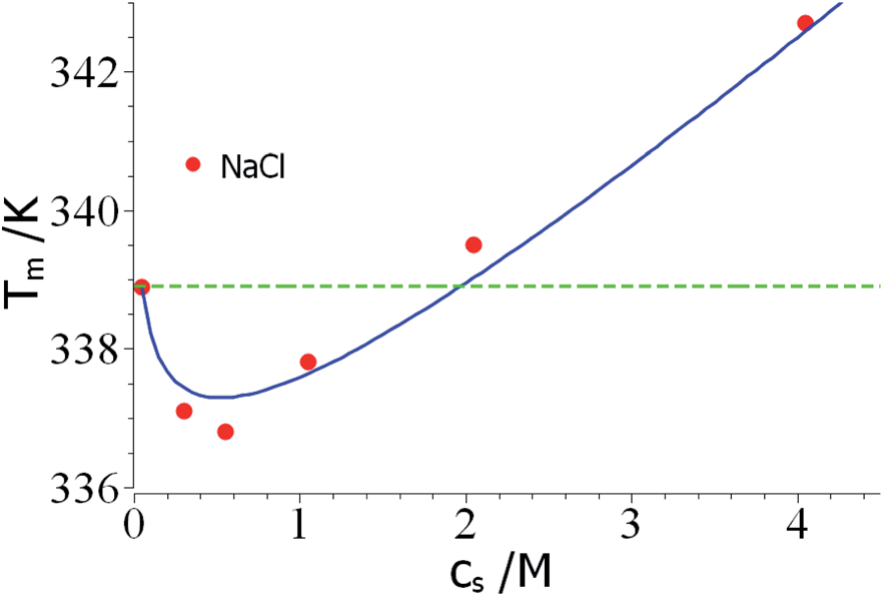
Reversal of *T*_m_ through a competition of counterion release and preferential hydration. The data marked by red points have been measured by Senske *et al.* for the unfolding of RNase A at pH = 5 in presence of an increasing concentration of NaCl.^16^ The solid line marks the fit by theory with the parameters Δ*n*_ci_ = −0.60 and Δ*w* = −31.7 (eqn (23), the term quadratic in Δ*T* has been neglected). The green dashed line marks the temperature *T*^0^_m_. See text for further explanation.

As mentioned above, the combination of a negative Δ*n*_ci_ with a negative Δ*w* value should lead to a non-monotonic dependence of *T*_m_ on salt concentration. This effect is seen in a careful study of the unfolding of RNase A in NaCl solutions by Senske *et al.*^16^ These data have been taken using a 50 mM citrate buffer at pH = 5 and are hence not directly comparable to the data of Francisco *et al.*^18^ discussed above. Fig. 3 displays the data obtained for solutions with varying concentration of NaCl. Since the range of temperature is rather small, the term quadratic in Δ*T* in eqn (23) can be disregarded. The fit of the data is shown by the solid line in Fig. 3 and leads to Δ*n*_ci_ = −0.60 and Δ*w* = −31.7. At small salt concentrations, the logarithmic term in eqn (23) dominates the transition temperature. In this regime, it stabilizes the unfolded state which takes up ions from solution more easily at higher salt concentration. At higher salt concentration, the term linear in salt concentration in eqn (23) takes over and the unfolded state is now destabilized leading to a higher *T*_m_ again.

## Conclusions

A phenomenological model describing the unfolding transition of proteins has been presented. Within this model, the change of *T*_m_ with the solute concentration *c*_s_ is captured by two effects: (i) the change of the number of correlated counterions Δ*n*_ci_ during the unfolding transition, and (ii) the change of hydration expressed through the parameter Δ*w*. The latter parameter is not directly the number of water molecules released or taken up during transition but described the change of the free energy by the release or uptake of water (see the discussion of eqn (11)). The model can be cast in terms of the closed expression eqn (23) giving the free energy of unfolding in terms of the salt/solute concentration *c*_s_. The enthalpy Δ*H*_u_ can directly be related to the parameter Δ*w* by the Maxwell-relation eqn (7) leading to eqn (8) in which a new parameter dΔ*c*_p_/d*c*_s_ describes the direct dependence of Δ*H*_u_ on salt concentration. The model allows us to discuss the classical *m*-value in terms of these parameters (eqn (16)) and predicts that *m* is depending on temperature if the parameter dΔ*c*_p_/d*c*_s_ assumes a finite value. A first comparison with experimental data taken from literature shows the general validity of the model.

## Conflicts of interest

There are no conflicts to declare.

## Supplementary Material
